# Investigating the human Calcineurin Interaction Network using the πɸLxVP SLiM

**DOI:** 10.1038/srep38920

**Published:** 2016-12-15

**Authors:** Sarah R. Sheftic, Rebecca Page, Wolfgang Peti

**Affiliations:** 1 Department of Molecular Pharmacology, Physiology and Biotechnology, Brown University, Providence, RI, 02912, USA; 2 Department of Molecular Biology, Cell Biology and Biochemistry, Brown University, Providence, RI, 02912, USA; 3 Department of Chemistry, Brown University, Providence, RI, 02912, USA.

## Abstract

Ser/thr phosphorylation is the primary reversible covalent modification of proteins in eukaryotes. As a consequence, it is the reciprocal actions of kinases and phosphatases that act as key molecular switches to fine tune cellular events. It has been well documented that ~400 human ser/thr kinases engage substrates via consensus phosphosite sequences. Strikingly, we know comparatively little about the mechanism by which ~40 human protein ser/thr phosphatases (PSPs) dephosphorylate ~15000 different substrates with high specificity. The identification of substrates of the essential PSP calcineurin (CN) has been exceptionally challenging and only a small fraction has been biochemically confirmed. It is now emerging that CN binds regulators and substrates via two short linear motifs (SLiMs), the well-studied PxIxIT SLiM and the LxVP SLiM, which remains controversial at the molecular level. Here we describe the crystal structure of CN in complex with its substrate NFATc1 and show that the LxVP SLiM is correctly defined as πɸLxVP. Bioinformatics studies using the πɸLxVP SLiM resulted in the identification of 567 potential CN substrates; a small subset was experimentally confirmed. This combined structural-bioinformatics approach provides a powerful method for dissecting the CN interaction network and for elucidating the role of CN in human health and disease.

One of the primary cellular signaling mechanisms used to transmit information is phosphorylation, with ≥98% of all known phosphorylation events occurring on serine and threonine residues (ser, thr). As such, it is the opposing activities of more than 428 ser/thr kinases and only ~40 ser/thr phosphatases (PSPs; see Table S1 for abbreviations) that ensures signaling pathway fidelity. While ser/thr kinases recognize their substrates using specific phosphosite consensus sequences, PSPs act on phosphosites that share little or no sequence similarity making the identification of substrates using sequence alone challenging^1^. As a result, our understanding of the role of PSPs in systems wide investigations has been severely limited.

Calcineurin (CN; Protein Phosphatase 2B or 3) is a ubiquitously expressed Ca^2+^/calmodulin activated PSP^2^. It is composed of a catalytic CNA subunit, which includes a calmodulin-binding and an auto-inhibitory domain, constitutively bound to a regulatory CNB subunit that binds 4 Ca^2+^ ions^2^. Increases in the cytoplasmic levels of Ca^2+^ activate CN, which then catalyzes the dephosphorylation of a plethora of substrates critical for a diverse set of biological processes including development, learning and memory^3^. However, in spite of its established role in these processes, thus far only ~50 CN substrates have been experimentally confirmed; this likely represents only a small fraction of the actual CN interaction network based on its cellular distribution and abundance^4^. Clearly, new approaches for identifying CN substrates are needed.

One process in which CN plays a critical role is in the activation of T-cells^5^. CN activation results in the dephosphorylation of the NFAT family of transcription factors (Nuclear Factor of Activated T-cell; NFATsc1-c4), resulting in their import into the nucleus and the induction of genes that activate T-cells^6^,^7^. A molecular understanding of how CN dephosphorylates its target substrates with high specificity in both space and time is only now beginning to emerge.

The efficient dephosphorylation of NFATs and other substrates by CN is not mediated by its recognition of specific phosphosite consensus sequences^8^,^9^,^10^. Rather, recent studies have shown that the NFATs bind directly to CN using two distinct short linear motifs (SLiMs), the “PxIxIT” and the “LxVP” motifs. These motifs were originally defined based on their conservation within the NFAT family (Fig. 1A) and function to tether CN near the NFAT phosphosites, which, in some cases, can be dozens of residues away from the SLiM sequences themselves^9^,^11^,^12^. The mechanism by which PxIxIT motifs bind CN is well established (PxIxIT residues bind an extended hydrophobic pocket present on the catalytic CNA subunit) and has led to a precise definition of this SLiM^13^,^14^,^15^. In contrast, a detailed understanding of how LxVP motifs engage CN is just developing. The first crystal structure of a CN-LxVP complex revealed that LxVP motifs bind CN using a second hydrophobic pocket that is ~30 Å from the PxIxIT binding site and is located at the CNA/B dimer interface^13^. Unexpectedly, the structure also revealed that this binding pocket is identical to that of the well-known immunosuppressants cyclosporin A and FK-506. Thus, in addition to providing a molecular explanation of how immunosuppressants prevent NFAT dephosphorylation (they block LxVP containing substrates from binding CN), this observation also provides the proof-of-principle that drugs and small molecules that bind directly to SLiM interaction pockets, such as the LxVP binding pocket, are potent, specific inhibitors of PSPs.

A molecular description of how multiple distinct PxIxIT and LxVP SLiMs bind CN will also lead to a comprehensive definition of these SLiM sequences, which can then be used to identify novel CN substrates based on sequence alone, something that thus far has been unachievable due to the lack of conservation of CN phosphosites^10^. The identification of these novel interactors will then lead to the discovery of new processes that are regulated by CN and, as a consequence, greatly expand CN protein interaction networks. While this approach has been successful for the PxIxIT SLiM in yeast^16^, a lack of structural data has not allowed the LxVP SLiM to be defined to an accuracy suitable for such a genome wide analysis. Furthermore, the location of the LxVP binding pocket for LxVP-containing substrates (versus inhibitors) is still not fully resolved. Namely, while multiple studies suggest that LxVP-containing substrates and inhibitors bind the same pocket at the CNA/B interface, a study using only the CNA subunit suggested that LxVP-containing substrates may also bind near the PxIxIT binding pocket^17^. Both issues have hampered the identification of novel CN substrates. Here, we combine structural biology and biochemistry to establish a structure-based definition of the LxVP SLiM and molecularly describe how LxVP-containing substrates bind CN. We then leverage this information with bioinformatics to obtain genome wide insights into CN function and tested our predictions on a small subset of targets experimentally. This analysis resulted in the identification of nearly 600 novel potential CN substrates and significantly expands the potential physiological roles of CN in both health and disease.

## Results

### The NFATc1 LxVP SLiM is ^384^DQYLAVP ^390^

**Figure 1:**
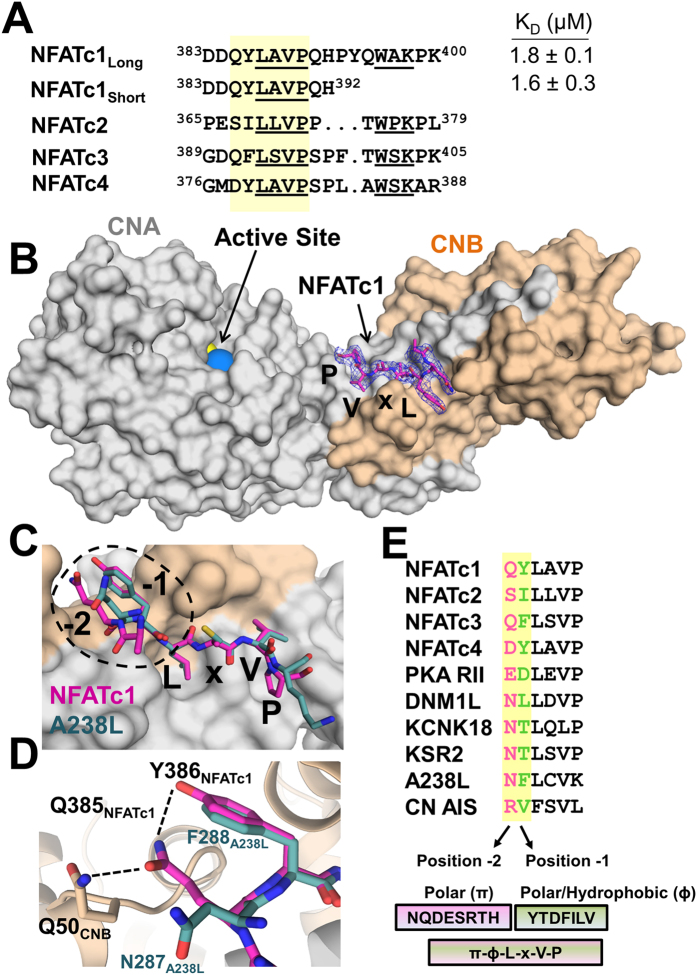
Structure of Calcineurin in complex with NFATc1 LxVP. (**A**) Sequence alignment of the NFATc1-c4 LxVP sequences and constructs used in this study. (**B**) 2.6 Å crystal structure of the CN:NFATc1_LxVP_ complex. NFATc1 LxVP peptide (pink sticks); CNA (grey surface) and CNB (beige surface). The active site metals Zn^2+^ and Fe^3+^ are shown as yellow and blue spheres respectively. Electron density of the NFATc1 LxVP peptide visible residues (^384^DQYLAVP^390^) shown as blue mesh (2F_o_-F_c_, σ = 1.0). **C)** Overlay of NFATc1 QYLaVP (pink sticks) and A238L NFLcVK (green sticks) motifs bound to CN. (**D**) Hydrogen bond network between Gln385_NFATc1_, Tyr386_NFATc1_ and Gln50_CNB_. (**E**) Alignment of the 10 experimentally confirmed LxVP motifs in CN binding proteins. The -2 (pink) and -1 (green) positions of all sequences are highlighted and were leveraged to generate the expanded 6-residue SLiM.

To determine how LxVP-containing substrates bind CN, we determined the 2.6 Å crystal structure of the CN:NFATc1_LxVP_ complex (CNA-CNB-NFATc1_LxVP;_^383^DDQYLAVPQHPYQWAKPK^400^) (Fig. 1B; Table S2). Electron density was present for NFATc1_LxVP_ SLiM residues ^384^DQYLAVP^390^ (Fig. 1B, S2A). The absence of electron density for NFATc1_LxVP_ residues 391–400 suggests that these residues remain flexible upon complex formation. To verify that only NFATc1_LxVP_ SLiM residues ^384^DQYLAVP^390^ are required for CN binding, we used isothermal titration calorimetry (ITC). Both peptides (^383^DDQYLAVPQHPYQWAKPK^400^ and ^383^DDQYLAVPQH^392^) bind CN with statistically identical K_D_’s, 1.8 ± 0.1 and 1.6 ± 0.3 μM, respectively (Fig. S1; Table S3). These K_D_s are similar to those reported for other LxVP motifs^13^ and confirms that the residues visible in the CN:LxVP_NFATc1_ crystal structure define the NFATc1 LxVP sequence that is necessary and sufficient for CN binding.

The NFATc1_LxVP_ SLiM, which buries ~950 Å^2^ of solvent accessible surface area, binds CN in an extended conformation to an expansive hydrophobic pocket located at the CNA/B interface (Fig. 1B). This is the same pocket occupied by LxVP-containing inhibitors^13^,^18^. As expected, the NFATc1 residues that interact most extensively with CN are Leu387_NFATc1_ and Val389_NFATc1_. Leu387_NFATc1_ binds into a deep hydrophobic pocket formed by CN residues Trp352_CNA_, Phe356_CNA_ and Val119_CNB_ while Val389_NFATc1_ is anchored by a comparatively shallower hydrophobic pocket formed by Trp352_CNA_, Pro344_CNA_, Leu343_CNA_, Try341_CNA_ and Leu123_CNB_ (Fig. 1C, S2B). The canonical ‘P’ in the LxVP sequence, Pro390_NFATc1_, binds a third shallow hydrophobic pocket defined by residues Trp352_CNA_, Pro344_CNA_ and Leu343_CNA_ (Fig. 1C, S2B). While the backbone of Ala388_NFATc1_, the ‘x’ in the LxVP sequence, forms a hydrogen bond with the sidechain of Trp352_CNA_, its side chain makes no interactions with CN. This explains the lack of a specific amino acid requirement in this position.

### The PxIxIT and LxVP SLiM binding sites do not overlap

Our structure shows that all known LxVP-containing CN interacting proteins (substrates, regulators, inhibitors) bind the same hydrophobic pocket on CN (Fig. 1C). This contrasts with a recent study which suggested that the NFATc1 peptide ^383^DDQYLAVPQHPYQWAKPK^400^ binds the CN PxIxIT, and not LxVP, binding pocket^17^. This raised the intriguing possibility that this SLiM sequence might bind to multiple, distinct pockets on a single enzyme, something that was previously thought impossible. To test this, we used ITC. Our data shows that the NFATc1_LxVP_^383^DDQYLAVPQHPYQWAKPK^400^ peptide binds weakly to CNA_1–348_ (~2-fold more weakly than to CNA/B). However, the core NFATc1_LxVP_ peptide, ^383^DDQYLAVPQH^392^, which binds CNA/B with the same affinity as the longer peptide, fails to bind CNA (Fig. S1, Table S3). This demonstrates that the weak interaction observed between NFATc1_LxVP_^383^DDQYLAVPQHPYQWAKPK^400^ and CNA is not due to an interaction with its LxVP sequence, but instead is due to non-specific interactions with ^393^PYQWAKPK^400^, the residues that are C-terminal to the LxVP motif. This confirms that the LxVP motif in NFATc1 binds, as our structure demonstrates, only the LxVP binding pocket.

### Defining the LxVP SLiM: πɸLxVP

NFATc1-c4 LxVP sequences, which are highly but not perfectly conserved, bind CN with distinct affinities^19^. Previous reports suggested that these differences were due to the variable number of residues between the LxVP motif and a second conserved sequence known as the WxK motif (Fig. 1A; this motif, which is just C-terminal to the LxVP motif, was previously hypothesized to bind directly to CN)^19^. Our structure and ITC data show that while the NFATc1 LxVP motif (^387^LAVP^390^) binds directly to CNA/B, the NFATc1 WxK motif (^396^WAK^398^) does not. This result is fully consistent with a previous NMR study which showed that no chemical shift perturbations are observed for Trp396_NFATc1_ in the presence of CNA/B^9^.

Thus, these data suggest that the binding affinity differences between the NFATs for CN originate from residues N-terminal to and/or including the LxVP motif. The residues immediately preceding the NFATc1-c4 LxVP motifs are similar, but not identical. First, the residues immediately N-terminal to the ‘L’ of the LxVP motif are always hydrophobic (i.e., Tyr386 in NFATc1, Ile387 in NFATc2; Fig. 1A). This suggests they may bind CN using a similar mechanism. Comparing the CN:NFATc1_LxVP_ complex with CN:A238L shows that Tyr386_NFATc1_ overlaps perfectly with Phe288_A238L_ (Fig. 1C). This structural conservation confirms, as previously suggested, that the LxVP motif is more accurately defined as ɸLxVP, where ɸ represents a hydrophobic residue^19^,^13^.

Second, Tyr386_NFATc1_ (ɸ residue) is part of a newly identified 3-residue hydrogen bond network (Fig. 1D) between the ɸLxVP peptide and CNB. The network is centered on the sidechain of Gln385_NFATc1_, which forms hydrogen bonds with both Tyr386_NFATc1_ and Gln50_CNB_. To date, 10 LxVP motifs in CN interacting proteins have been biochemically confirmed (Fig. 1E). However, in all 10 motifs, the sidechains of amino acids in the -2 position ([-2]-ɸ-L-x-V-P), such as Gln385_NFATc1,_ function as hydrogen bond donors/acceptors. These data demonstrate that a residue capable of forming polar interactions in the -2 position is a defining feature of this motif. This is further supported by the observation that mutating the ɸ residue to an amino acid capable of hydrogen bonding in two CN substrates, DNML1^20^ and NFATc2^19^, both of which have polar residues in the -2 position, enhances CN binding. Together, these data demonstrate that the LxVP SLiM is most accurately defined as πɸLxVP, where π and ɸ represent a polar and hydrophobic residue, respectively (Fig. 1E).

### Identifying novel, potential CN substrates using the expanded πɸLxVP SLiM

**Figure 2:**
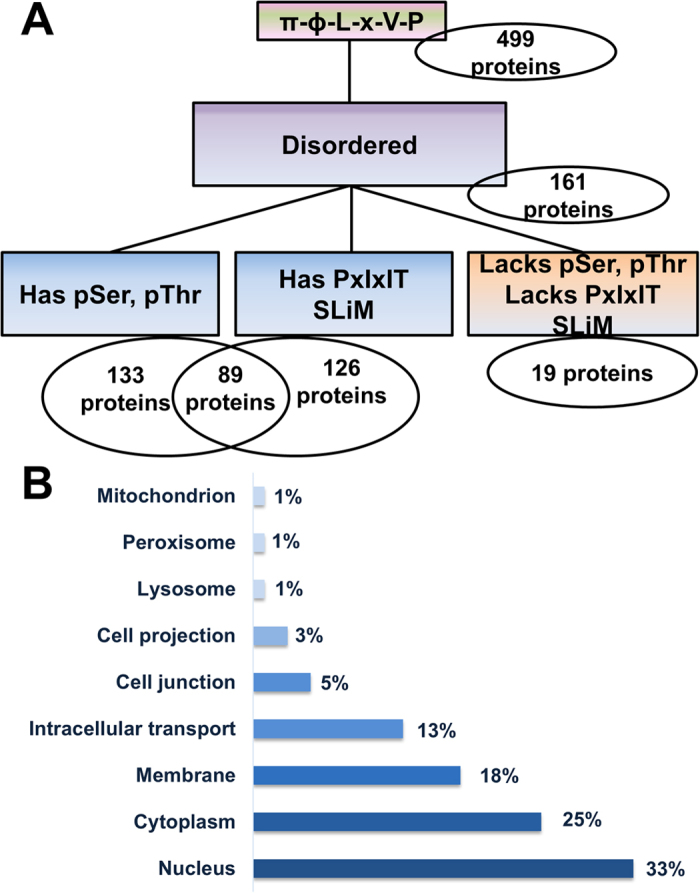
The correct LxVP SLiM is a πɸLxVP motif. (**A**) Flow chart of the πɸLxVP motif search using Scanprosite, IUPRED and Uniprot to identify CN substrates. Scanprosite was used to probe the *Homo Sapiens* database for hits containing the expanded LxVP sequence generated from the experimental 3D-structure of the CN:NFATc1_LxVP_ complex in combination with 10 other biochemically confirmed LxVP sites in CN interacting proteins (pink/green box). IUPRED was subsequently used to select only hits contained in intrinsically disordered regions (IDRs) based on a cutoff of 0.4 for IDR propensity (purple box). UniProt and Scanprosite were used to apply filters for hits containing pSer/pThr residues and putative PxIxIT sites respectively (blue boxes). A subset of 19 hits do not contain pSer/pThr residues and also lack putative PxIxIT sites (orange box). (**B**) Cellular distribution of the 89 proteins identified that contain putative LxVP and PxIxIT motifs in IDRs as well as experimentally confirmed pSer/pThr residues (UniProt).

The ability to use the LxVP SLiM to identify new CN interacting proteins was previously hindered by conflicting models of LxVP binding^13^,^17^ and the lack of specificity of the motif. Our structure of CN in complex with its most well-studied substrate, NFATc1, overcomes these limitations as it confirmed that all structurally characterized LxVP sequences bind the same pocket on CN (Fig. 1C). Our structure also led to the discovery that the NFATc1 LxVP SLiM is more specific than previously thought and defined as πɸLxVP (Fig. 1D,E). A search of the Uniprot human database using this expanded SLiM, which includes all residues that have polar donor/acceptor sidechains in the -2 position as well as all residues in the 10 experimentally confirmed LxVP sites in the -1 position (π-ɸ-LxVP: [NQDESRTH]-[YTDFILV]-L-x-V-P), results in 518 hits in 499 sequences (Fig. 2A).

To maximize the likelihood that these πɸLxVP-containing proteins represent bona fide CN substrates, we applied three additional filters. The first filter ensured that all identified motifs were present in intrinsically disordered proteins (IDPs) or regions (IDRs; IUPRED ≥ 0.4) of their corresponding proteins. This is because all known SLiMs are intrinsically disordered and, thus far, all experimentally confirmed LxVP SLiMs have been found in IDRs. This IDP filter reduced the number of hits by more than 65%, resulting in 173 motifs in 161 proteins (Fig. 2A; 4 proteins contained multiple πɸLxVP motifs). The second filter ensured that the identified proteins had been previously observed to be phosphorylated on one or more ser/thr residues (Uniprot). This reduced the number of proteins to 133 (Fig. 2A). In parallel, a third filter was used to show that 126 of the 161 original πɸLxVP-IDR containing proteins also contained the CN-specific PxIxIT SLiM (Fig. 2A). By taking the intersection of these two sets, we identified that 89 distinct proteins contain both a pSer/pThr residue and a PxIxIT SLiM. This provides compelling support that, at minimum, 89 (55%) of the 161 proteins with expanded πɸLxVP motifs in IDPs or IDRs are highly likely CN substrates. Interestingly, 37 proteins contained PxIxIT SLiMs but lacked pSer and/or pThr residues, indicating that this subset of hits might be modulators of CN activity but are not CN substrates, or that their phosphorylation/dephosphorylation sites are currently unknown.

An analysis of the cellular distribution of these 89 likely CN substrates revealed that the highest fraction (33%) is found in the nucleus (Fig. 2B), consistent with the known roles of CN to dephosphorylate transcription factors and DNA binding proteins that are critical for the G1-S transition of the cell cycle^21^,^22^. The large distribution of πɸLxVP-proteins in the membrane (18%) and intracellular transport (13%) is also consistent with the prominent roles of CN in ion transport and vesicle trafficking, respectively^23^,^24^.

### Redefining the minimal four residue LxVP core sequence

**Figure 3:**
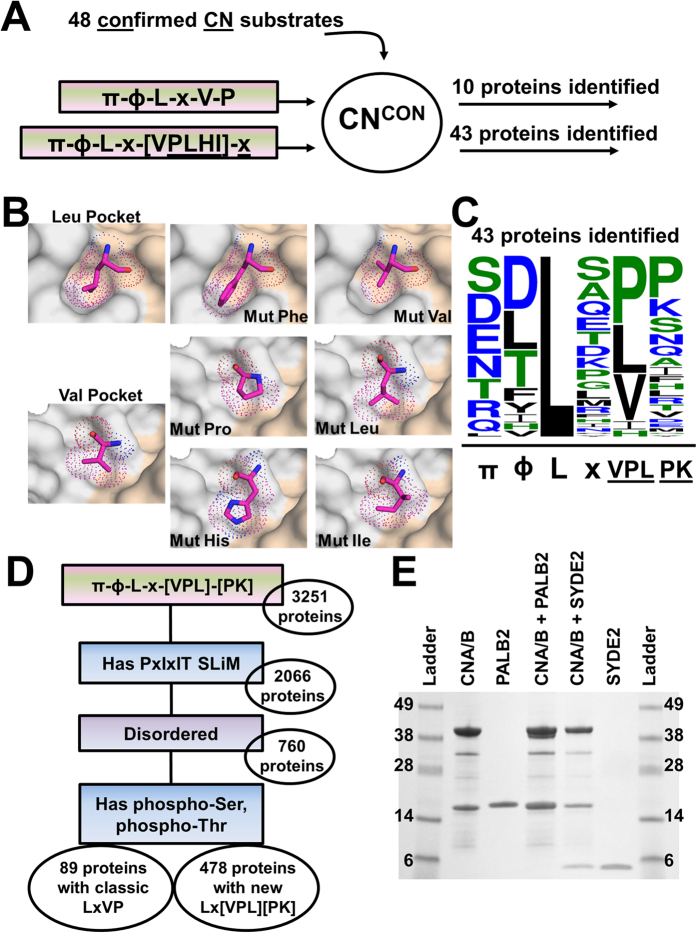
Redefining the LxVP core. (**A**) The expanded ([NQDESRTH]-[YTDFILV]-L-x-[VPL]-x → π-ɸ-L-x-VPLHI-x) SLiM was used in a search of 48 confirmed CN interacting proteins in humans (CN^CON^). (**B**) The Leu and Val core residue binding pockets (CNA, grey surface; CNB, beige surface). Residues are shown as pink sticks and dots to demonstrate Van-der-Waals distances. **C)** Weblogo of 43 CN^CON^ proteins based on a π-ɸ-L-x-VPLHI-x search. Residue symbols are weighted based on probability. (**D**) The expanded 6 residue [NQDESRTH]-[YTDFILV]-L-x-[VPL]-[PK] → π-ɸ-L-x-VPL-PK SLiM was used as a search sequence in the *Homo Sapiens* database in ScanProsite. The resulting hits were subjected to 3 filters, i) presence of a putative PxIxIT SLiM (blue box), ii) propensity for intrinsic disorder (purple box) and iii) presence of experimentally confirmed pSer, pThr residues (blue box). (**E**) Direct interaction of CN with PALB2 and SYDE2.

In parallel, we compiled a database of the 48 proteins that have been experimentally demonstrated to interact directly with CN (referred to as CN^con^ – confirmed CN interactors; to date, no LxVP SLiM has been identified for ~80% of these interactors; Table S4). We then searched for a πɸLxVP SLiM in each protein. Interestingly, only 20% of CN^CON^ returned hits on our expanded πɸLxVP SLiM (Fig. 3A), showing that the expanded πɸLxVP definition is too conservative and fails to detect CN interactors that have similar, yet also binding compatible sequences. Because all CN structures determined to date (free and CN complexes), including that described here, confirm that the conformation of the LxVP pocket is rigid, the structure of the CN-based ‘lock’ is viewed as conformationally fixed while the SLiM-based ‘key’ is viewed as flexible. Therefore, we used the structure of the CN:πɸLxVP_NFATc1_ complex to identify amino acids that can be readily accommodated in the Leu, Val and Pro hydrophobic binding pockets. Critically, this structure-based approach is not biased in regards to the interaction strength of the individual LxVP sequence, i.e. all predicted sequences are most likely binding competent. This is important, as different K_D_’s for the same SLiM binding pocket are likely important for driving distinct signaling events.

The Leu binding pocket is very narrow and fails to accommodate other amino acids without significant changes in the sidechain rotomer conformations (Fig. 3B). Thus we conclude that the Leu residue of the LxVP motif is conserved in the majority of CN interactors. The Val binding pocket, in contrast, is comparatively shallow and readily accommodates a Pro, Ile, His, or Leu (Fig. 3B). Indeed, the confirmed LxVP motif of KCNK18 (NTLqLP) contains a Leu in the Val position of this SLiM (Fig. 1E)^25^. The Pro pocket is also very shallow, allowing for a broad range of substitutions at this site. In the CN:A238L complex (NFLcVK; Fig. 1C) the Lys residue, which lies across the Pro pocket, is nearly completely solvent exposed. Thus, we allowed this position to be any residue in a search against CN^CON^. Searching CN^CON^ using this structure-based motif ([NQDESRTH]-[YTDFILV]-L-x-[VPLIH]-x → π-ɸ-L-x-[VPLHI]-x) returned 43 of the 48 proteins (Fig. 3A, Table S4). A sequence alignment (Weblogo, V.3.4) of the 43 identified motifs identified favored residues in the Val and Pro positions of the πɸLxVP SLiM (Fig. 3C)^26^. This data immediately shows that most residues can be accommodated in the Pro position with only a slight preference for Pro and Lys, which are also experimentally, structurally confirmed residues. Interestingly, Val, Leu and Pro are nearly equally probable in the Val position, with a much lower population of Ile and His. By combining the structural data with the CN^CON^ derived data, we derived the optimized definition for this SLiM: [NQDESRTH]-[YTDFILV]-L-x-[VPL]-[PK] → π-ɸ-L-x-[VPL]-[PK]. Searching for this motif using ScanProsite yielded 3251 human proteins. After employing the same secondary filters used previously (requires a PxIxIT site, the motif is present in an IDP or IDR and the protein has experimentally confirmed pSer/pThr residues), the list condenses to 567 proteins, 89 of which contain a canonical ‘π-ɸ-LxVP’ motif and 478 that contain a ‘π-ɸ-L-x-VPL-PK’ motif (Fig. 3D).

The cellular distribution of these new potential CN substrates remains virtually unchanged compared to that identified using the conservative SLiM definition, with the largest fraction of CN interacting proteins having key roles in the nucleus (Fig. S3). An analysis of these 567 proteins also showed that the preferred average distance of the first pSer/pThr from the π-ɸ-L-x-VPL-PK site is within 50 residues (Fig. S4). Notably, there is a preference for phosphosites to be C-terminal to the SLiM (80%), similar to what is observed for the canonical CN substrate, protein kinase A (PKA) subunit RII. In contrast, there are fewer predicted interactors with phosphosites that are N-terminal of the SLiM (20%), which is where the phosphosites are located in NFATs.

### Expanding the CN protein interaction and functional networks

**Figure 4:**
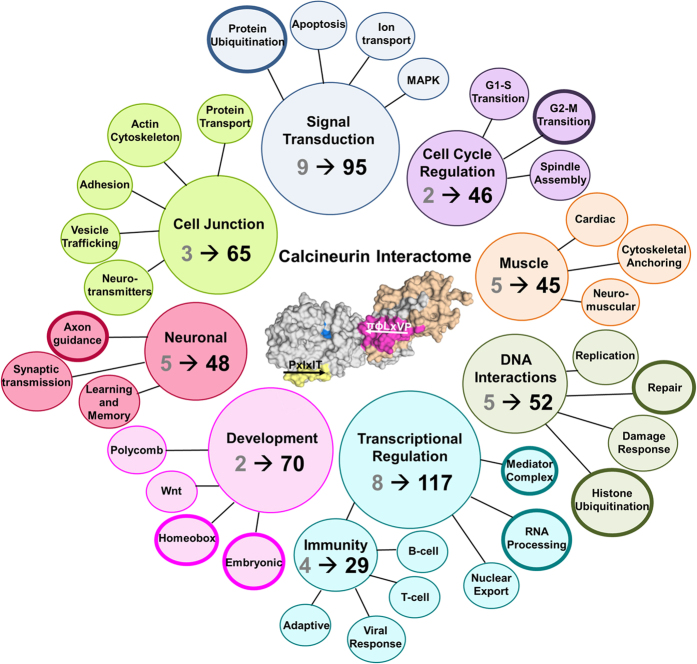
Human Calcineurin Interactome. Change in the functional distribution of the CN^CON^ and 567 predicted CN interacting proteins. Grey numbers correspond to CN^CON^ proteins of the designated function. Black numbers correspond to the number of predicted proteins associated with the designated function. Bold outlines correspond to novel CN roles.

Our study identified novel potential CN interactors, which both expanded known and suggested completely novel physiological functions for CN. An overview of these functions is shown in Fig. 4, covering roles from immunity to signaling to transcription.

#### A-kinase anchor proteins (AKAPs)

CN has been widely studied in the context of its interaction with A-kinase anchoring protein 79, AKAP79 (aka AKAP5), and its subsequent role in synaptic plasticity^27^,^28^. This analysis identifies additional AKAPs that are predicted to bind CN, which, like AKAP79, function to co-localize CN with PKA and protein kinase C. These new AKAPs include CRBG3/CRYBG3/vlAKAP (πɸLxVP: ^1027^SFLKVP^1032^) and AKAP12 (πɸLx-VPL-PK: ^197^QLLTVK^202^). AKAP12 has been shown to bind Ca^2+^/Calmodulin via its 3 positively charged domains (PCDs)^29^. Due to its homology with AKAP5/AKAP79, AKAP12 is also predicted to interact with CN via these domains^30^. Consistent with this, our predicted AKAP12 πɸLx-VPL-PK site (^197^QLLTVK^202^) is between PCD1 (residues 171–187) and PCD2 (residues 297–317), which, incidentally, is also the location of the predicted AKAP12 PxIxIT site (^284^PTSPVT^289^).

#### JNK signaling

Our data suggest that CN has a prominent role in multiple signal transduction pathways. In particular, we show that CN likely plays a critical role in the JNK signaling pathway by directly modulating the activity of mitogen activated kinases MAP3K7/TAK1 (πɸLxVP: ^420^NILDVP^425^; activates JNK and p38 pathways), MAP3K10 (πɸLxVP: ^634^SYLSVP^639^; activates the JNK pathway), MAP3K11 (πɸLx-VPL-PK: ^659^RDLQPP^664^; activates the JNK pathway) and MAP3K19 (πɸLx-VPL-PK: ^640^NYLDLK^645^). Similarly, CN also dephosphorylates the transcription factors ELK1^31^ and c-JUN^32^, which themselves are phosphorylated by MAPK8–10 of the JNK pathway. Taken together, this data provides compelling evidence that the CN PSP and JNK kinases act antagonistically *in vivo* and are critical for the regulation of the (pro)apoptotic response following cellular stress.

#### DNA Repair

This study also suggested new roles for CN during the cell cycle and in particular, DNA repair (Fig. S5). Specifically, a cluster of proteins implicated in the breast cancer 2 (BRCA2) mediated DNA repair mechanism are identified. PALB2 (πɸLx-VPL-PK: ^220^SVLIPP^225^) is an essential regulator of DNA double strand break repair whose phosphorylation leads to the recruitment of BRCA2 (πɸLx-VPL-PK: ^3378^DYLRLK^3383^) and RAD51 (not predicted to bind CN)^33^,^34^. By dephosphorylating PALB2 and BRCA2, CN is able to modulate the DNA damage response. CN further moderates BRCA2 activity in the context of transcriptional repression due to its predicted direct interaction with the BRCA2-interacting transcriptional repressor (EMSY: πɸLx-VPL-PK: ^1045^QVLAVK^1050^)^35^. Taken together, this work establishes that CN has a prominent role in modulating the DNA damage response and may be a primary mediator of DNA repair (Fig. S5).

As DNA repair is a novel role for CN, we experimentally confirmed the interaction between PALB2 and CN. PALB2_178–295_, which includes the predicted PxIxIT and LxVP SLiMs, interacts directly with CN, as shown by pulldown and ITC assays (Fig. 3E, S1; Table S3).

#### Ubiquitination

Our analysis also suggested that CN is important for protein ubiquitination, a novel role for CN. Namely, CN is predicted to bind and regulate the E3 ubiquitin protein ligase CBLB (πɸLx-VPL-PK: ^803^DLLIPP^808^; Cbl Proto-Oncogene B) and the E3 ubiquitin protein ligase adaptor protein CBL (πɸLx-VPL-PK: ^540^RDLPPP^545^), both of which negatively regulate T-cell and B-cell receptor pathway activity^36^,^37^. CN is also suggested to bind the E3 ubiquitin ligase HERC2 (πɸLxVP: ^3350^SYLGVP^3355^), an enzyme that is an integral regulator of the DNA damage response to ionizing radiation^38^. Lastly, RNF8 (πɸLx-VPL-PK: ^255^RILRLK^260^), which fulfills a key role in the DNA damage response via histone ubiquitination as it recruits repair machineries, including TP53BP1/BRCA1, and activates ATM kinase^39^, is also a potential CN interactor.

#### Transcription

This work also expands the CN-transcription factor interactome beyond NFATs. Our analysis suggests that CN regulates the phosphorylation state of more than 100 distinct transcription factors that affect multicellular organism development and function from embryogenesis through adulthood, including Homeobox regulating proteins Cux1 (πɸLx-VPL-PK: ^739^TILTPK^744^), MSL1 (πɸLxVP: ^471^SVLAVP^476^) and SIX4 (πɸLxVP: ^674^DLLSVP^679^). Furthermore, CN may play a larger role in general transcriptional regulation through interactions with components of the Mediator complex, responsible for transcription from RNA polymerase II genes, MED1 (πɸLxVP: ^904^NTLGVP^909^), MED12 (πɸLx-VPL-PK: ^1637^QLLPLP^1642^) and MED14 (πɸLx-VPL-PK: ^1183^NILLLP^1188^).

Lastly, CN’s importance for neuronal function is well established. Here we identify 48 potential interactors that correlate CN dephosphorylation with neuronal activity and highlight an unexplored role of CN in axon generation and guidance (Fig. 4). We demonstrate, by pulldown and ITC, that the synapse defective protein 1 homolog 2 (SYDE2) (residues 232–289; πɸLx-V-P: ^235^RVLSVP^240^) binds CN (Fig. 3E, S1; Table S3). In *C. elegans,* SYD-1 (SYDE2 homolog) has been shown to have an important role in neuronal polarization, culminating in the formation of axons and dendrites^40^.

## Discussion

Despite the clear biological importance of CN, elucidating a human genome wide interaction network for this essential enzyme has been exceptionally challenging and thus far only a small subset of proteins has been experimentally confirmed to be CN substrates (CN^CON^, Table S4). This is because, in contrast to protein tyrosine phosphatases (PTPs), identifying PSP substrates has been comparatively difficult, as the approaches for identifying substrates of PTPs, including substrate trapping mutants and selective inhibitors, are much less developed for PSPs^41^. Thus, identifying and defining novel SLiMs that are specific for distinct PSPs, such as the PxIxIT SLiM in CN^16^, provides a key avenue to overcome these challenges. Here we leveraged our novel structural insights to accurately define the LxVP SLiM, π-ɸ-L-x-VPL-PK, and then used this motif to identify novel, potential CN interacting proteins/substrates. Indeed, the structure-based SLiM analysis presented here can be thought of as a molecular ruler, in which the rigid enzyme (CN) reduces the primary sequence space of the interacting SLiM. The advantage of this technique is that all identified π-ɸ-L-x-VPL-PK SLiMs will be binding competent. The disadvantage is that it likely only selects for strong(er) binders. However, in a cellular environment where multiple substrates compete for the same CN binding site, the stronger interactors are likely often the preferred interactors. Our analyses resulted in the discovery of 89 predicted interactors that contain a conservative π-ɸ-L-x-V-P SLiM and 478 predicted interactors that contain the structure-based, comprehensive π-ɸ-L-x-VPL-PK SLiM (Fig. 3D). We confirmed the quality of our prediction method, by randomly selecting a couple of predicted interactors and experimentally confirming their interaction with CN.

The effectiveness of our study is furthermore immediately apparent as novel πɸLxVP sites were identified in well-established, bone fide CN substrates, including ^2156^TLLSPK^2161^ in Cain/Cabin1 and ^678^RDLMPK^683^ in Dynamin1^42^,^43^. We also identified new πɸLxVP sites in proteins identified as potential CN interactors in the BioGRID3.4^44^,^45^ and STRING10.0^46^ protein interaction databases, including the membrane receptor KLRG2 (πɸLxVP: ^149^RFLKVP^154^), the ser/thr kinase SIK3 (πɸLx-VPL-PK: ^472^SLLQPP^477^) and the transcriptional regulator MEF2A (πɸLxVP: ^212^TDLTVP^217^). Critically, however, our study also identified hundreds of new potential CN interactors (Fig. 4). The analysis identified CN interactors that have functions previously not associated with CN, including protein ubiquitination (HERC2, CBLB, CBL) and DNA repair (PALB2, BRCA2). It also substantially expanded that number of interactors that are essential for well-established functions, such as transcription (SIX4, MED12, among others). This immediately suggests a plethora of expanded and novel functions of CN in key signaling pathways and critical cellular processes in the cell. Over the next years, it will be exciting to test these predictions further and to leverage the information for the analysis of the functional consequences of CN dephosphorylation of its novel substrates in cell physiology.

## Material and Methods

### Expression and Purification

CN was expressed and purified as previously described^13^. Briefly, CNA (residues 1–370), CNB (residues 1–169) was subcloned into a bicistronic p11 vector containing an N-terminal His_6_-TEV tag and expressed in *E. coli* after growth to an OD_600_ = ~0.8 and induction with 1 mM IPTG for 18 hours at 18 °C. Cells were harvested by centrifugation at 5,400 × g at 4 °C for 15 minutes and stored at −80 °C. Cells were lysed using a high pressure cell homogenizer (Avestin) and lysates were centrifuged at 45,400 × g at 4 °C for 60 minutes. Purification was initiated by Ni^2+^-NTA purification followed by TEV cleavage. CN was further purified by anion exchange chromatography (HiTrap Q HP), followed by size exclusion chromatography (SEC, Superdex 75 26/60) using 10 mM Tris pH 7.5, 100 mM NaCl, 1.0 mM DTT, 1.0 mM CaCl_2_ as the final buffer. Peptides (^383^DDQYLAVPQHPYQWAKPK^400^; ^383^DDQYLAVPQH^392, 116^ESPRIEITS^124^) were purchased from Biosyn Inc. (≥95% purity; Lewisville, TX). PALB2 (residues 178–295) and SYDE2 (residues 232–289) were cloned into pJ411 vectors containing an N-terminal His_6_-TEV tag (DNA2.0: Newark, CA). PALB2 and SYDE2 were expressed in *E. coli* after growth to an OD_600_ = ~1.0 and induction with 1.0 mM IPTG for 20 hours at 18 °C. Cells were harvested and lysed as described above. Briefly, purification was initiated by Ni^2+^-NTA purification followed by TEV cleavage. PALB2 and SYDE2 were further purified by SEC (Superdex 75 26/60) using 20 mM Tris pH 7.5, 100 mM NaCl, 0.5 mM TCEP as the final buffer.

### Crystallization and structure determination

The CN:NFATc1_LxVP_ complex (concentration: ~9 mg/ml) formed crystals in 0.1 M HEPES pH 7.0 and 15% (w/v) PEG4000 at 25 °C. Crystals were obtained using the hanging drop vapor diffusion method (24-well Linbro plate, Hampton), with 2 μl drops containing a 1:2 ratio of precipitant solution to protein and 500 μl of precipitant solution in the reservoir. Crystals were harvested under oil and immediately flash frozen in liquid N_2_. Data were collected at the Stanford Synchrotron Radiation Lightsource (BL9-2) at SLAC National Accelerator Laboratory. Crystals of the CN:NFATc1_LxVP_ complex formed in space group P2_1_2_1_2_1_, with unit cell dimensions *a* = 57.67 Å, *b* = 106.04 Å, *c* = 111.92 Å, and α*βγ* = 90°. Data were indexed, integrated and scaled with the autoxds suite^47^. The structure of the CN:NFATc1_LxVP_ complex was determined to 2.6 Å by molecular replacement using a CN heterodimer molecule^13^ (CNA/B; PDB ID 4F0Z as the search model). The final model of the CN:NFATc1_LxVP_ complex was obtained using iterative rounds of refinement in PHENIX^48^ and model building in Coot^49^. The asymmetric unit contains one copy of the CN:NFATc1_LxVP_ complex. CNA residues 1–10, CNB residues 1–4 and NFATc1 LxVP residues 391–400 were not visible in the electron density map and thus were not modeled. The final structure refined to a R factor of 19.5% (R_free_ = 23.9%). 100% of residues are in the allowed region of the Ramachandran diagram. Structure validation and stereochemistry analysis was performed with Molprobity^50^.

### Isothermal Titration Calorimetry

ITC experiments were performed at 25 °C using a VP-ITC microcalorimeter (Malvern). All protein samples were equilibrated in ITC buffer (20 mM Tris pH 7.5, 100 mM NaCl, 1.0 mM CaCl_2_, 0.5 mM TCEP). NFAT peptides, PALB2 and SYDE2 were titrated into CNA/B. Titrant (10 μL per injection for NFAT peptides and 12 μL per injection for PALB2 and SYDE2) was injected into the sample cell over a period of 20 seconds with a 250 second interval between titrations to allow for complete equilibration and baseline recovery. 27 (10 μL) or 21 (12 μL) injections were delivered during each experiment, and the solution in the sample cell was stirred at 307 rpm to ensure rapid mixing. Data were analyzed with a one set binding site model, based on the 1:1 stoichiometry observed in the crystal structure, using Origin 7.0 (OriginLab).

### Pulldown Assay

CNA/B was purified as described, without His_6_-tag cleavage. All proteins were prepared in Pull Down Buffer (50 mM Tris pH 8.0, 500 mM NaCl, 1 mM CaCl_2_). 2 mL of 5 μM CNA/B was applied to 1.5 mL Ni^2+^-NTA resin (GE Healthcare). 1.5 mL of 10 μM purified PALB2 or SYDE2 was added to the CNA/B containing resin and incubated at 4 °C for 45 minutes to allow for complex formation. Following incubation, beads were washed with 10 mL of pull down buffer. Complex containing fractions were eluted with 50 mM Tris pH 8.0, 500 mM NaCl, 500 mM Imidazole. 10 μL fractions were pooled with 10 μL 2x SDS loading buffer and boiled at 90 °C for 5 minutes. Samples were analyzed using SDS-PAGE and stained with Coomassie Brilliant Blue.

### Bioinformatics

ScanProsite^51^ was used to identify additional CN interacting proteins that contain an LxVP SLiM. Definitions of the search sequences were based on the experimental 3-dimensional CN:NFATc1_LxVP_ complex structure and experimentally confirmed LxVP motifs (Fig. 1E). Filter 1: Disorder prediction via IUPRED^52^,^53^ was used to ensure that identified πɸLxVP SLiMs are in disordered protein regions, as it is well recognized that SLiMs are only identified in IDP or IDRs (IUPRED ≥0.4). Filter 2: To increase the likelihood of identifying CN substrates, a PxIxIT SLiM (ScanProsite)^51^ in IDR (IDPRED ≥0.4) filter was applied. Two PxIxIT definitions were used: [P]-x-x-x-[IV]-[TDH] NFAT/PVIVIT-like^54^; or [P]-x-x-x-x-[IV]-[TDH] AKAP79-like using an extended CN binding pocket^14^; [IV]-[TDH] were based on experimentally confirmed sites^13^,^15^,^55^,^56^. Based on the focus on CN *substrates*, only proteins with experimentally confirmed phosphorylated Ser/Thr residues (using UniProt) were further considered as the final filter. Phosphosite distances relative to the N and C-terminal end(s) of the π-ɸ-L-x-VPL-PK motif were also determined and analyzed. A cutoff of 9 residues was used for phosphosites C-terminal to the SLiM. A distance of 9 residues is sufficient to cover the ~30 Å gap to the active site^13^. A cutoff of 20 residues was used for phosphosites N-terminal to the SLiM. Hits were cross-validated via literature searches and/or interaction databases^45^.

## Additional Information

**Accession Code:** The structure factors and coordinates for the CN-NFATc1 LxVP complex have been deposited with the Protein Databank with accession number 5SVE.

**How to cite this article:** Sheftic, S. R. *et al*. Investigating the human Calcineurin Interaction Network using the πɸLxVP SLiM. *Sci. Rep.* **6**, 38920; doi: 10.1038/srep38920 (2016).

**Publisher's note:** Springer Nature remains neutral with regard to jurisdictional claims in published maps and institutional affiliations.

## Supplementary Material

Supplementary Information

Supplementary Information
