# Supplementary material for: Investigating the human Calcineurin Interaction Network using the πɸLxVP SLiM
Source: Sci Rep. 2016 Dec 15;6:38920. doi: 10.1038/srep38920 (PMC5156906; doi:10.1038/srep38920)
Supplement: Supplementary Information [file srep38920-s1.pdf]

Supplementary Data:

Investigating the human Calcineurin Interaction Network using the πϕLxVP SLiM

Sarah R. Sheftic[1], Rebecca Page[2] and Wolfgang Peti[1,3*]

Table S1. Abbreviations.

Abbreviations	
Ca^{2+}	calcium ion
IDP	intrinsically disordered protein
IDR	intrinsically disordered region
ITC	Isothermal titration calorimetry
IUPRED	Programs used to predict of the structural propensity of proteins
PSP	protein ser/thr phosphatase
SLiM	short linear motif
SLiM sequences	
LxVP	SLiM also specific for CN (L, leucine; V, valine)
ϕLxVP	SLiM in which ϕ represents a hydrophobic amino acid
πϕLxVP	SLiM in which π represents a polar amino acid
PxIxIT	SLiM specific for CN (P, proline; x, any amino acid, I, isoleucine; T, threonine)
WxF	sequence motif just C-terminal to the LxVP sequence in NFATs
Gene/proteins	
AKAPs	A-kinase anchor proteins
BRAC1/BRAC2	breast cancer 1 or 2 protein
CBLB	Cbl Proto-Oncogene B
CN	calcineurin
CNA	the A-subunit of calcineurin
CNB	the B-subunit of calcineurin
Cux1	Cut Like Homeobox 1
DNML1	dynamin 1 like protein; a CN substrate
ELK	transcription factor
EMSY	BRAC2 interacting transcriptional repressor
HER2C	HECT And RLD Domain Containing E3 Ubiquitin Protein Ligase 2
JNK	c-jun N-terminal kinase
MAP3K	Mitogen activate protein kinase kinase kinase
MED	Mediator Complex Subunit
MSL1	Male Specific Lethal 1 Homolog
NFAT/NFATc1-c4	nuclear factor of activated T-cells c1-c4
PALB2	partner and localizer of BRAC2
PCD1/PCD2	positively charged domains 1 and 2
PKA subunit RII	cAMP-Dependent Protein Kinase A regulatory subunit-II A
PKC	Protein kinase C

SIX4	Sine Oculis Homeobox Homolog 4
SYDE2	Synapse Defective Protein 1 Homolog 2
TP53BP1	Tumor suppressor p53-binding protein 1

Table S2. Data collection and refinement statistics

	CNA_{1-370},CNB_{1-169}; NFAT LxVP peptide[a]
**Data collection**	
Space group	P 2_1 2_1 2_1
Cell dimensions	
a, *b*, *c* (Å)	57.7, 106.0, 111.9
α, β, γ (°)	90, 90, 90
Resolution (Å)	39.04 - 2.60 (2.71 - 2.60) *
R_{merge}	0.115 (0.805)
Mean I / σI	4.7 (1.7)
Completeness (%)	99.6 (97.8)
Redundancy	7.3 (7.2)
$CC_{1/2}$	0.998 (0.773)
**Refinement**	
Resolution (Å)	39.03 – 2.60
No. reflections	21785
R_{work} / R_{free}	0.19/0.24
No. atoms	
Protein	4333
Ligand/ion	58
Water	52
B-factors	
Protein	47.40
Ligand/ion	53.30
Water	41.60
R.M.S. deviations	
Bond lengths (Å)	0.004
Bond angles (°)	0.597
Ramachandran	
Outliers (%)	0.00
Allowed (%)	4.4
Favored (%)	95.6

[a]Data was collected from a single crystal
*Values in parentheses are for highest-resolution shell.

Table S3: ITC data

CN	NFAT Peptides	K_D (μM)	ΔH (kcal*Mol^{-1})	TΔS (kcal*Mol^{-1})
(A) 1-370 (B) 1-169	^{383}DDQYLAVPQHPYQWAKPK^{400}	1.8 ± 0.1	-19.5 ± 0.2	-11.7 ± 2.2
(A) 1-370 (B) 1-169	^{383}DDQYLAVPQH^{392}	1.6 ± 0.3	-3.1 ± 0.9	4.8 ± 0.8
(A) 1-348	^{116}ESPRIEITS^{124}	4.1 ± 0.1	-1.5 ± 0.2	5.8 ± 0.2
(A) 1-348	^{383}DDQYLAVPQHPYQWAKPK^{400}	2.4 ± 0.2	-1.1 ± 0.1	-6.5 ± 0.1
(A) 1-348	^{383}DDQYLAVPQH^{392}	No binding	n/a	n/a
	**New Substrates**			
(A) 1-370 (B) 1-169	^{178}PALB2^{275}	3.1 ± 1.6	-2.6 ± 1.4	5.0 ± 1.2
(A) 1-370 (B) 1-169	^{232}SYDE2^{289}*	2.6 ± 0.08	-3.2 ± 1.8	4.5 ± 1.8

Average of ≥3 ITC experiments for all data points.
*Average of 2 ITC experiments.

Table S4. Proteins in CN^{CON}

	Uniprot ID	Protein		Uniprot ID	Protein
**1**	P49418	AMPH	**25**	P11137	MAP2
**2**	Q92934	BAD	**26**	P10636	MAPT
**3**	P15056	BRAF (RAF)	**27**	Q9NP98	MYOZ1
**4**	Q13936	CACNA1C	**28**	Q9NPC6	MYOZ2
**5**	P27824	CANX	**29**	Q8TDC0	MYOZ3
**6**	Q9Y2V2	CARHSP1	**30**	P12036	NEFH
**7**	O95180	CAC1H	**31**	O95644	NFATC1
**8**	P02489	CRYAA	**32**	Q13469	NFATC2
**9**	P49674	CSNK1E (CK1)	**33**	Q12968	NFATC3
**10**	Q05193	DNM1	**34**	Q14934	NFATC4
**11**	O00429	DNM1L	**35**	Q08499	PDE4D
**12**	P50570	DNM2	**36**	Q13522	PPP1R1A
**13**	P11532	DMD	**37**	Q9UD71	PPP1R1B
**14**	P19419	ELK1	**38**	P13861	PRKAR2A
**15**	Q9Y6I3	EPN1	**39**	P06400	RB1
**16**	P42566	EPS15	**40**	P53805	RCAN1
**17**	P21333	FLNA	**41**	Q14206	RCAN2
**18**	P42261	GRIA1	**42**	Q9UKA8	RCAN3
**19**	P49841	GSK3B (GSK)	**43**	P19634	SL9A1
**20**	P56537	IF6	**44**	O60641	SNAP91, AP180
**21**	P05412	JUN	**45**	Q8WYL5	SSH1
**22**	P13861	KAP2	**46**	O43426	SYNJ1
**23**	Q7Z418	KCNK18	**47**	Q5T7P8	SYT6
**24**	Q6VAB6	KSR2	**48**	P31629	Zep2

Figure S1.

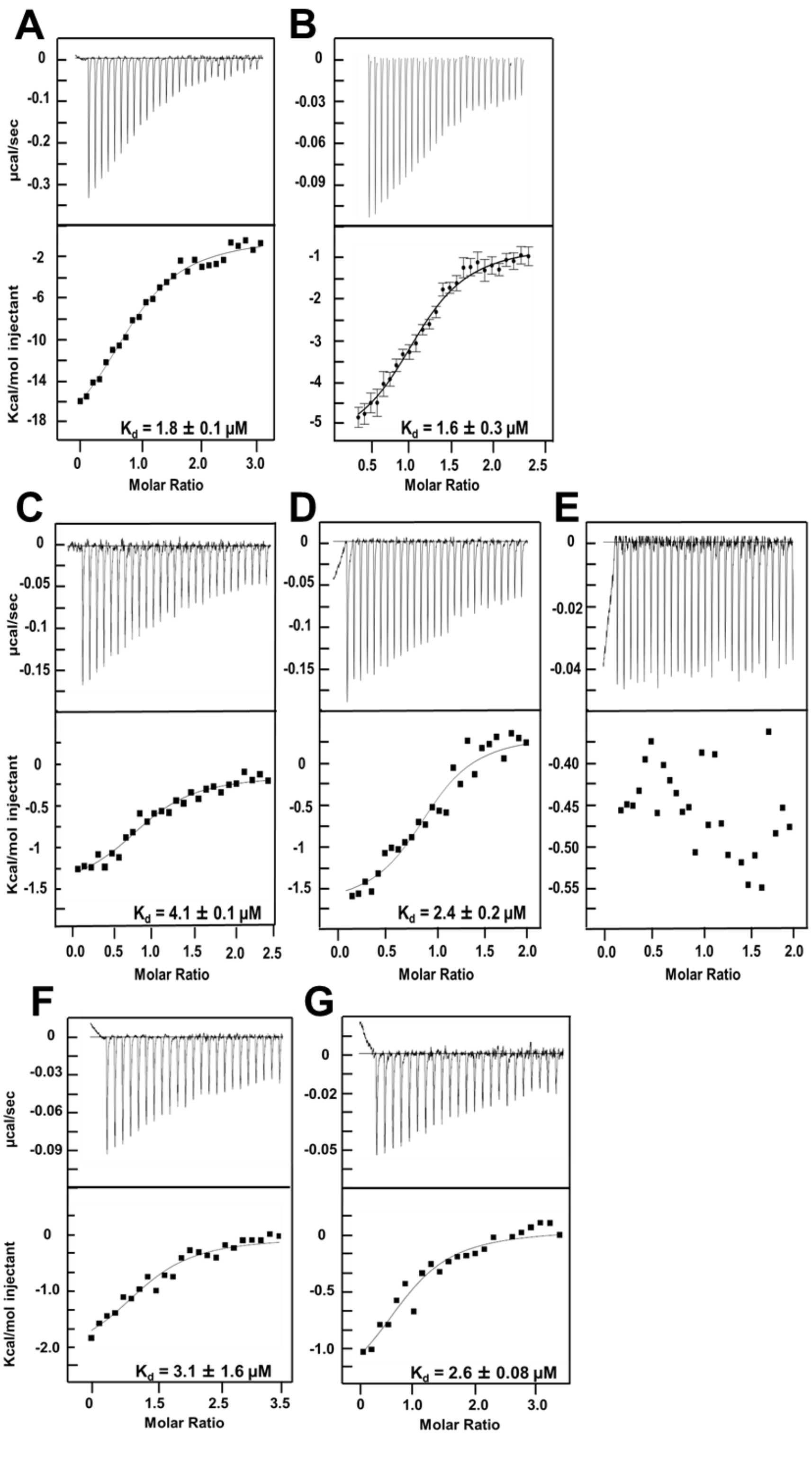
A
B
C
D
E
F
G
µcal/sec
Kcal/mol injectant
Molar Ratio
K_d = 1.8 ± 0.1 µM
K_d = 1.6 ± 0.3 µM
K_d = 4.1 ± 0.1 µM
K_d = 2.4 ± 0.2 µM
K_d = 3.1 ± 1.6 µM
K_d = 2.6 ± 0.08 µM

Figure S1. *NFATc1 binds to CN via a short πϕLxVP motif and requires CNA and CNB.* Isothermal titration calorimetry (ITC) data for CN (A: 1-370, B: 1-169) with NFATc1 substrates **(A)** LxVP$_{long}$ peptide: ^{383}DDQYLAVPQHPYQWAKPK^{400} and **(B)** LxVP$_{short}$ peptide: ^{383}DDQYLAVPQH^{392}. ITC data for CNA (1-348) with NFATc1 **(C)** PxIxIT peptide ^{116}ESPRIEITS^{124} **(D)** LxVP$_{long}$ peptide: ^{383}DDQYLAVPQHPYQWAKPK^{400} and **(E)** LxVP$_{short}$ peptide: ^{383}DDQYLAVPQH^{392}. ITC data for CN (A: 1-370, B: 1-169) with substrates **(F)** PALB2 protein (residues 178-275) and **(G)** SYDE2 protein (residues 232-289). Raw thermograms and the corresponding curve fits with a stoichiometry n=1 are shown. Thermodynamic parameters are listed in **Table S1**.

Figure S2.

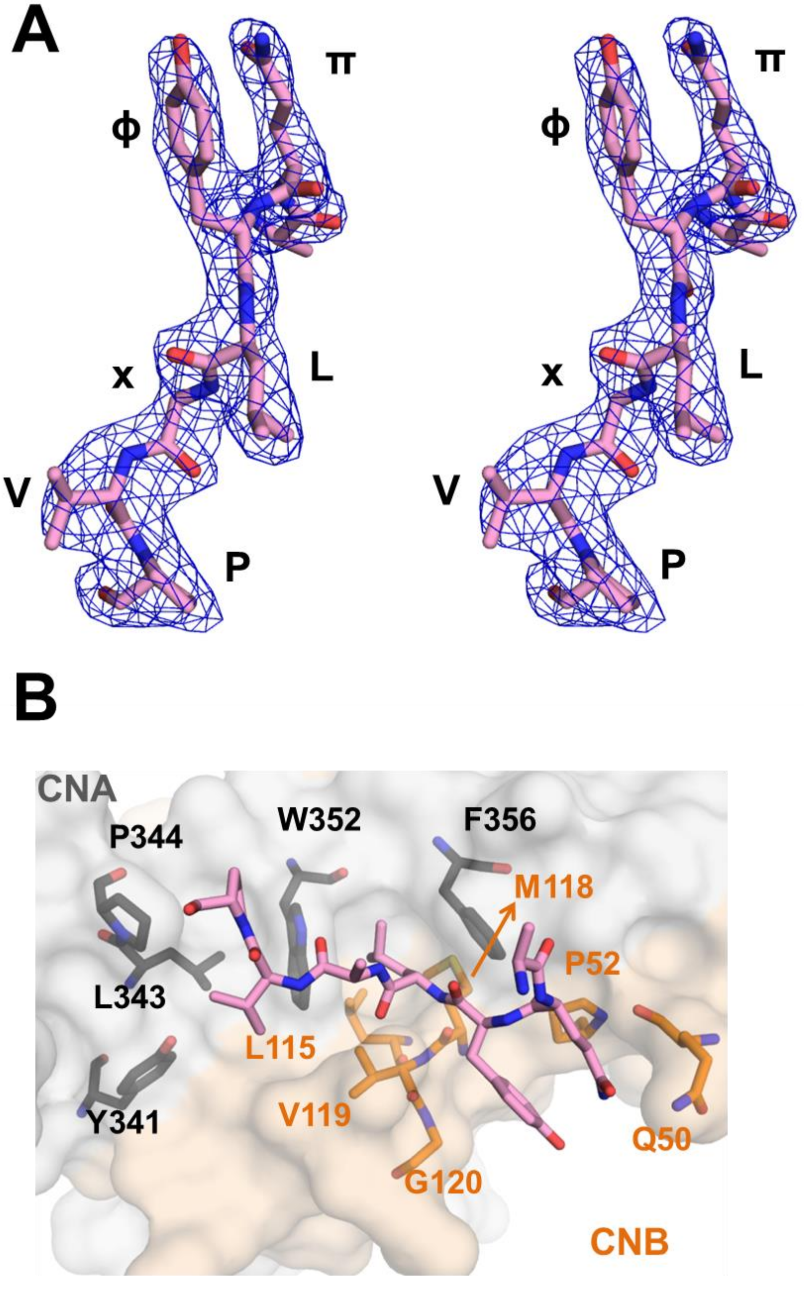

Figure S2. *NFATc1 πϕLxVP binding is mainly driven by hydrophobic, but also a few critical electrostatic interactions*. **A)** Stereo-view of the NFATc1 LxVP motif. Electron density of the NFATc1 LxVP peptide visible residues (^{384}DQYLAVP^{390}) shown as blue mesh ($2F_o$-F_c, σ = 1.0). **B)** Residues of CNA (dark grey sticks) and CNB (orange sticks) that mediate hydrophobic contacts with NFATc1 LxVP are shown.

Figure S3.

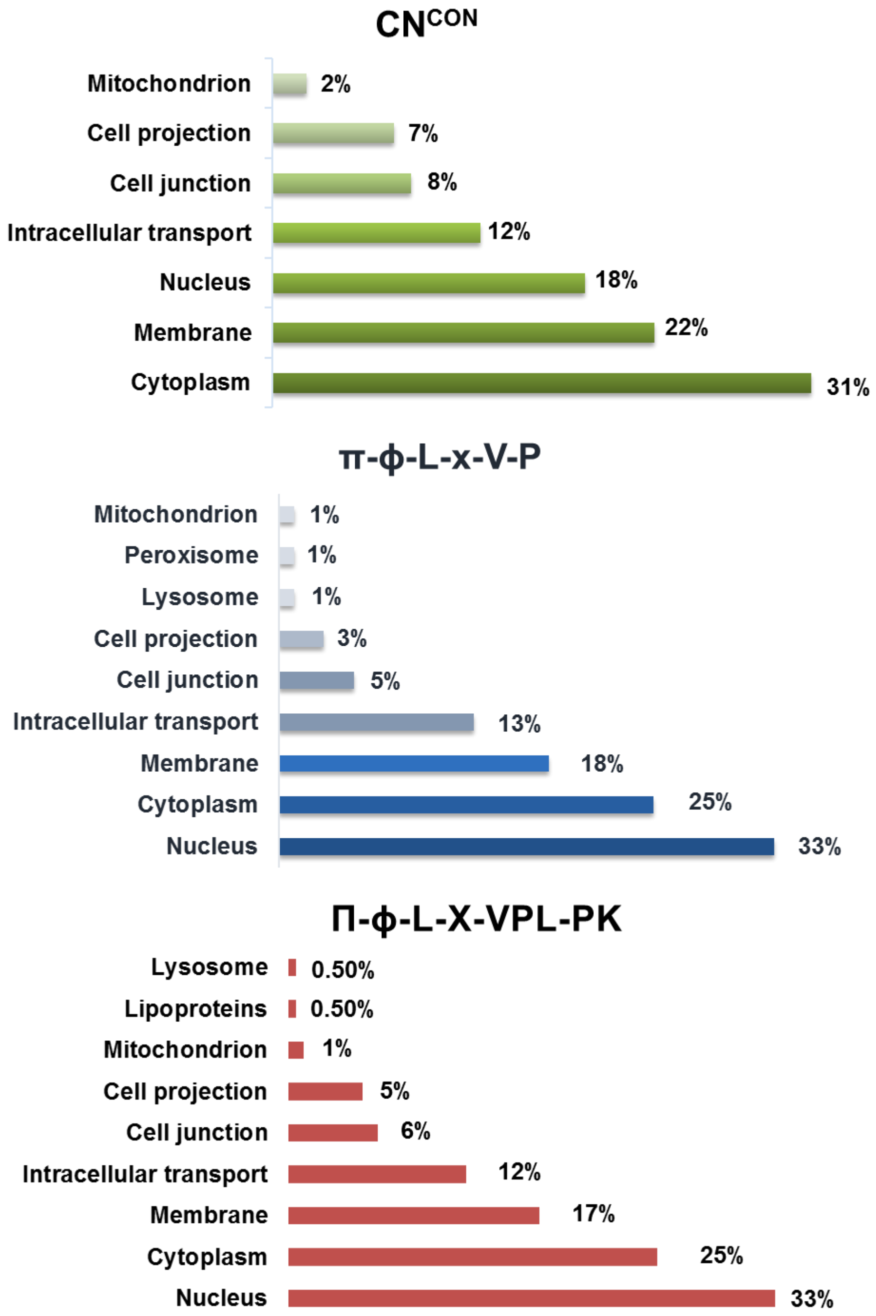

Figure S3. *The cellular distribution of CN substrates remains highly similar among all 3 databases*. Cellular distribution of the 48 proteins represented in CN^{CON} (green), the 91 proteins that contain putative π-ϕ-L-x-**V-P** motifs (blue) and the 565 proteins that contain putative π-ϕ-L-x-**VPL-PK** motifs (red). Proteins from the π-ϕ-L-x-**V-P** and π-ϕ-L-x-**VPL-PK** databases also contain putative PxIxIT SLiMs contained in IDRs and experimentally confirmed pSer/pThr residues (UniProt).

Figure S4.

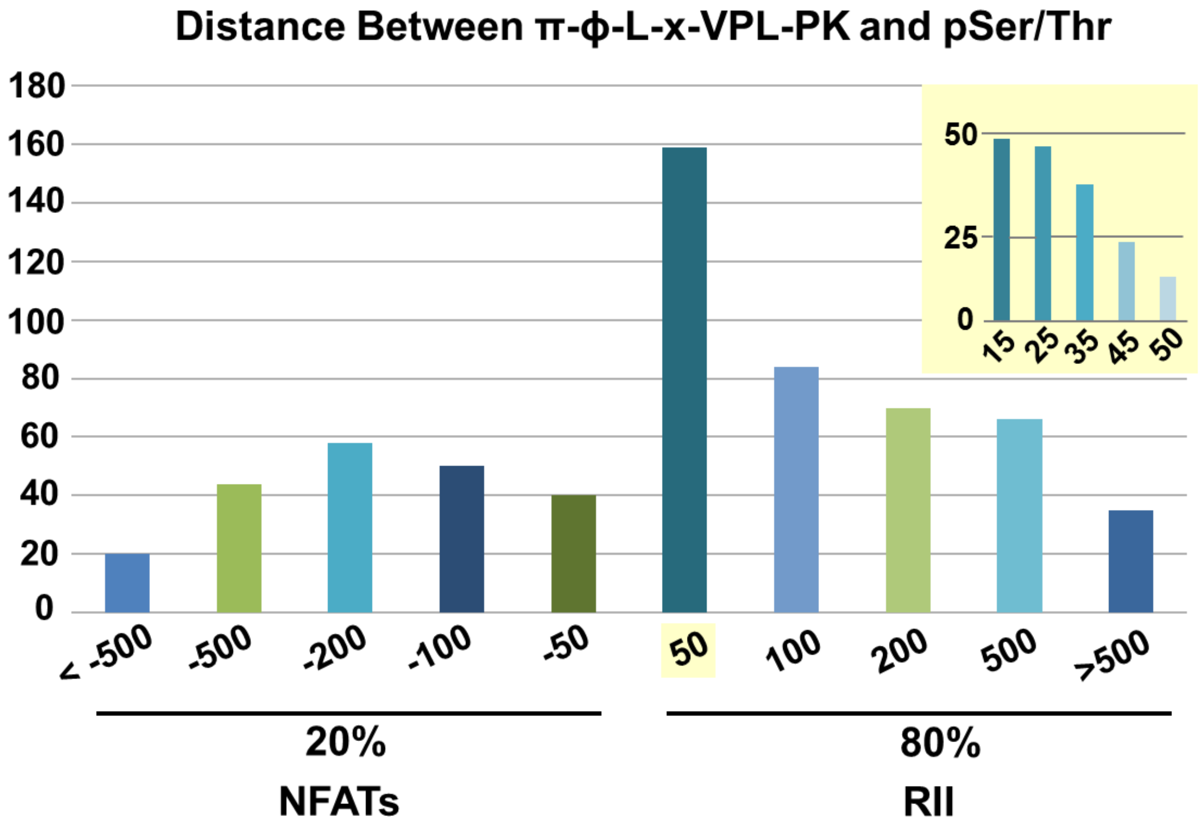

Figure S4. *Distribution of phosphosite distances from the π-ϕ-L-x-VPL-PK SLiM.* Distances (number of residues) between the nearest pSer/Thr residue from the predicted π-ϕ-L-x-VPL-PK SLiM of the interacting proteins. Phosphosites N- and C-terminal to the SLiM were considered. A minimum distance of 9 residues for C-terminal and 20 residues for N-terminal pSer/pThr sites were used as cutoffs based on the distance between the active site and the π-ϕ-L-x-VPL-PK SLiM binding site. For interacting proteins with multiple predicted π-ϕ-L-x-VPL-PK SLiMs, the pSer/pThr residues nearest to each motif were used for this analysis.

Figure S5.

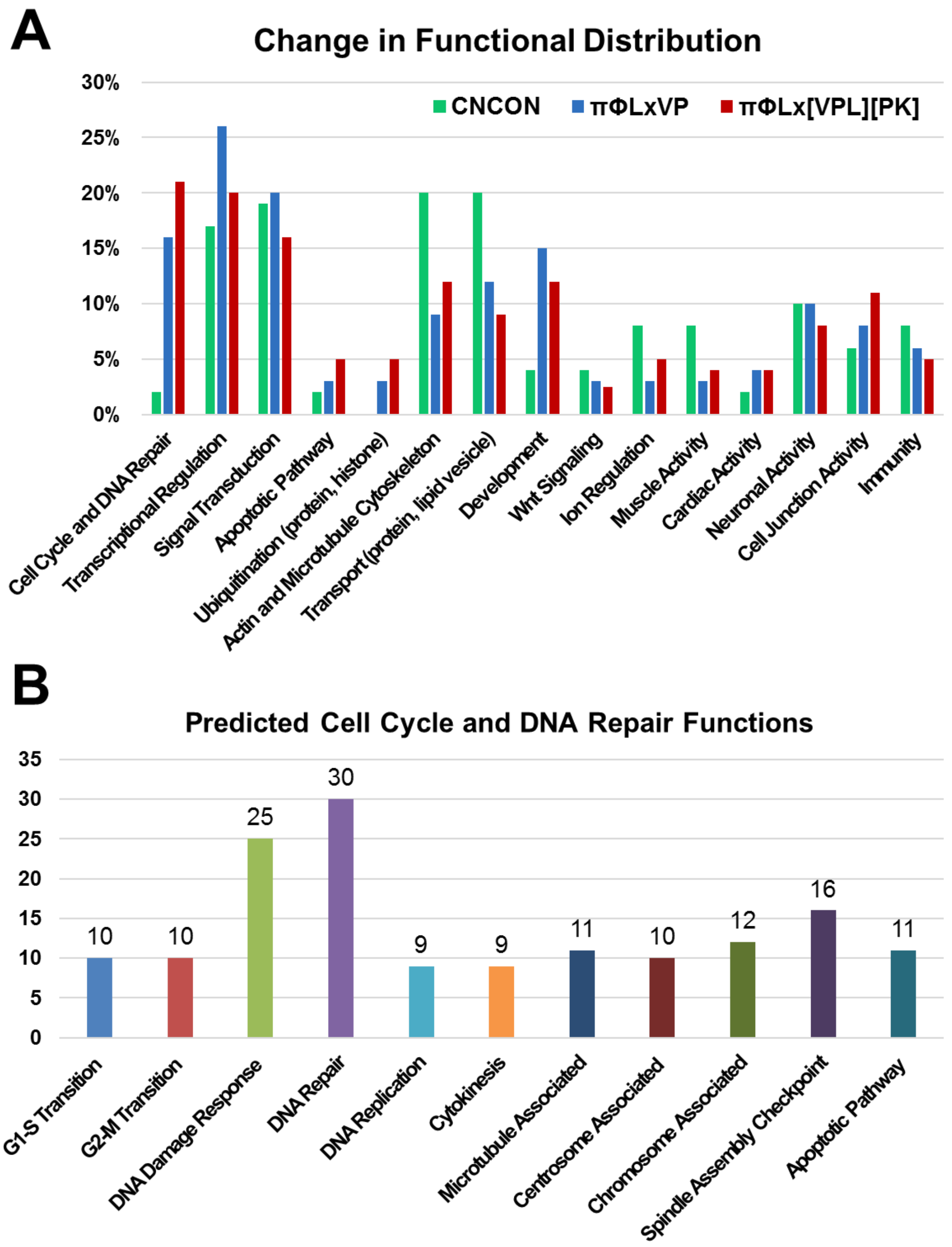

Figure S5. *Change in the functional distribution of CN activity among 3 databases.* **(A)** Comparison of the functional distribution of the proteins contained within CN^{CON} (green), the π-ϕ-L-x-**V-P** database (blue) and the π-ϕ-L-x-**VPL-PK** database (red). **(B)** Distribution of the predicted CN interactors that have roles in cell cycle regulation and DNA repair.
